# Comparative Evaluation of the Antihyperglycemic Potential of *Coccinia cordifolia* and *Gynura procumbens* Leaf Extracts: Integrated Phytochemical, In Vivo, Molecular Docking, and DFT Investigations

**DOI:** 10.1002/fsn3.72227

**Published:** 2026-08-10

**Authors:** A. J. M. Morshed, Sujan Kanti Das, Zobayed Islam, Mamuna Akter, Muhammad Abu Bakar, Mohammad Mostafa, Rasheda Akter, Tania Sharmin, Mohammad Helal Uddin

**Affiliations:** ^1^ Bangladesh Council of Scientific and Industrial Research (BCSIR) Chattogram Laboratories Chattogram Bangladesh; ^2^ Department of Pharmacy, Faculty of Biological Sciences University of Chittagong Chattogram Bangladesh; ^3^ Department of Applied Chemistry and Chemical Engineering University of Chittagong Chattogram Bangladesh

**Keywords:** antihyperglycemic activity, blood glucose level, *Coccinia cordifolia*, diabetes mellitus, *Gynura procumbens*, streptozotocin‐induced diabetes

## Abstract

Diabetes mellitus (DM) is a metabolic disorder defined by prolonged hyperglycemia due to deficient secretion or utilization of insulin. Although there exist various antidiabetic drugs, they are not devoid of side effects, are of low potency and expensive, thus necessitating the development of alternative natural antidiabetic remedies. The current investigation assessed the antihyperglycemic activity of *Coccinia cordifolia* and *Gynura procumbens* ethanolic leaf extracts on an STZ‐induced diabetic rat model, in conjunction with molecular docking and DFT calculations. Initial phytochemical investigations revealed the presence of alkaloids, flavonoids, phenolic, steroid compounds, saponins, and tannins in the extracts. Acute toxicity studies showed no toxicological signs when the extracts were administered up to 2 g/kg body weight. Administration of extracts orally for 14 days decreased fasting blood glucose (FBG) in a dose‐dependent fashion. At 2 g/kg body weight, the extracts of *G. procumbens* and *C. cordifolia* lowered the blood glucose by 55.74% and 52.68%, respectively, while glibenclamide, the standard drug, exhibited 61.96% reduction in blood glucose. From molecular docking, 2,5‐Dioxopyrrolidine‐1‐carboximidamide, 2,4‐Dimethylbenzo[h]quinoline, and actinobolin were established as the best bioactive compounds with higher binding affinities (−7.5 to −7.2 kcal/mol) compared to the known drug glibenclamide (−6.3 kcal/mol). In addition, favorable electronic stability and reactivity were found after conducting the DFT calculations. Overall, it can be concluded that the two plant extracts, especially *G. procumbens* , have promising antihyperglycemic effects and thus can act as good sources of bioactive compounds to be used as novel plant‐based antidiabetic drugs.

## Graphical abstract

Graphical abstract illustrating the overall experimental workflow and antidiabetic evaluation of *Coccinia cordifolia* and *Gynura procumbens* extracts. Plant materials were extracted and subsequently investigated through in vivo antidiabetic studies, density functional theory (DFT) analysis, and in silico molecular docking to identify potential bioactive compounds and elucidate their antidiabetic mechanisms.

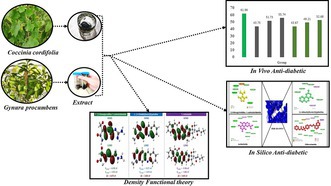

## 1 Introduction

Diabetes mellitus (DM) is one of the fastest‐growing noncommunicable diseases across the globe. It is defined as a condition of hyperglycemia caused by either reduced insulin production, defective insulin action, or a combination of both and associated with metabolic disorders in carbohydrates, lipids, and proteins. Persistent hyperglycemia leads to a variety of complications such as diabetic nephropathy, neuropathy, retinopathy, cardiovascular diseases, and stroke that affect the morbidity, mortality, and medical costs (Parveen et al. 2017). The International Diabetes Federation (IDF) estimates that more than 589 million adults had diabetes in 2025 and that the figure will significantly increase over the next decades, especially in the developing countries where there is limited access to good quality healthcare. Therefore, the development of new and effective treatment strategies for diabetes has become a very important issue at a global level (Zhou et al. 2022; Mamun et al. 2026; Sultana et al. 2025).

Present‐day pharmacological treatment of diabetes includes insulin administration and different oral hypoglycemic drugs, such as sulfonylureas, biguanides, thiazolidinediones, DPP‐4 inhibitors, SGL‐2 inhibitors, and GLP‐1 receptor agonists. Though these drugs show good efficacy in improving glycemic control, their long‐time administration is often accompanied by such unpleasant side effects as hypoglycemia, digestive system abnormalities, weight gain, liver damage, and low patient compliance. In addition, the growing economic cost of drug therapy hampers the availability of drugs in many developing countries. All these factors have led to increased interest in plant drugs as a potential alternative to conventional pharmacotherapy due to their wide chemical composition, multitarget pharmacological activity, and better safety profile (Al‐Rajhi, Bakri, et al. 2023a; Xue et al. 2025; Islam et al. 2025).

Plant drugs have been used for ages in traditional medical systems to treat diabetes and other metabolic disorders (Ajithkumar et al. 2020; Hermanto et al. 2019). Many secondary metabolites from plants such as flavonoids, phenolic acids, alkaloids, terpenoids, saponins, and tannins have been found to possess potent antihyperglycemic, antioxidant, anti‐inflammatory, and insulin‐sensitizing properties. Phytochemicals have been reported to be effective in managing diabetes using various mechanisms like promotion of insulin secretion, protection of pancreatic β‐cells from oxidative stress, peripheral glucose uptake, inhibition of carbohydrate hydrolytic enzymes, blocking of hepatic gluconeogenesis, and alteration of signaling mechanisms for glucose homeostasis. The multitarget approach of phytochemicals is different from many antidiabetic medications with a single molecular target mechanism (Islam et al. 2026; Devi et al. 2024).

Among medicinal plants that are commonly used for treatment of diabetes, there are two plants, which draw growing attention of the scientists due to diverse therapeutic properties of these plants. One of these plants is *Coccinia cordifolia* , which belongs to the family *Cucurbitaceae* and is commonly known as the perennial climbing herb. *Coccinia cordifolia* has been traditionally used for treatment of diabetes, inflammations, liver ailments, wounds, and gastrointestinal disorders in traditional medicine in South Asia. The preliminary studies on the phytochemicals contained in the plant have shown that the leaves of this plant are rich in various antioxidants and glucose‐lowering agents such as flavonoids, phenolics, triterpenoids, alkaloids, and glycosides (Al‐Rajhi, Qanash, et al. 2023b).

Likewise, *Gynura procumbens*, belonging to the *Asteraceae* family, is well‐known for being an important medicinal plant in Southeast Asia. The herb has numerous pharmacological actions, including antidiabetic, antihypertensive, antioxidative, anti‐inflammatory, hepatoprotective, antimicrobial, and anticancer effects. These biological actions are reported to be due to the high content of flavonoids, derivatives of chlorogenic acid, phenolics, steroids, and other active compounds found in this medicinal plant. Research shows that *G. procumbens* increases insulin sensitivity, inhibits glucose uptake from the intestines, strengthens antioxidant defense systems, and regulates several pathways controlling glucose homeostasis. However, there is no research on the relative effectiveness of *G. procumbens* compared with *C. cordifolia* under the same experimental conditions (Mestry et al. 2017; Xu et al. 2024; Liu et al. 2022).

While individual studies have separately investigated the antidiabetic activities of both of these medicinal herbs, there are still some critical aspects to be considered. Previous studies conducted have mainly been done by investigating only one type of plant and not compared in order to see which one shows better antihyperglycemic effects. Also, most investigations conducted are mainly concerned with in vivo pharmacological testing rather than using computational methods that can aid in identifying potential bioactive compounds and determining their interactions at the molecular level with targets of therapeutic importance in diabetes (Al‐Rajhi, Qanash, et al. 2023b; Meem et al. 2026; Algariri et al. 2013). Furthermore, few studies provide information on electronic and chemical reactivity properties of major phytoconstituents present in them. Hence, it would be beneficial if both types of methods were used to find new antidiabetic drug candidates along with understanding the mechanism of action behind them. With regard to the current increasing global incidences of diabetes, the shortcomings associated with the currently existing treatments, and the ongoing need for low‐cost plant‐based drugs, the comparison of these medicinal plants becomes highly rational from both scientific and clinical aspects (Xie et al. 2022; Raghav et al. 2022; Islam et al. 2026). The identification of their effectiveness, safety, and mode of action may contribute to the discovery of new phytotherapeutic candidates that can supplement the conventional treatment of diabetes. In this connection, this study sought to compare the antihyperglycemic effects of ethanolic leaf extracts of *Coccinia cordifolia* and *Gynura procumbens* in streptozotocin‐induced diabetic rats. Apart from the phytochemical investigation, acute toxicity test, and antihyperglycemic activity in vivo, molecular docking of the compounds against a target related to diabetes was done, followed by density functional theory (DFT) calculation of electronic structures and reactivity of the promising candidates.

## 2 Materials and Methods

### 2.1 Plant Materials

The fresh leaves of *C. cordifolia* and *G. procumbens* were collected from different areas of Chattogram. The plant species were identified by the botany research division BCSIR Chattogram. After collection, the leaves of *Coccinia cordifolia* and *Gynura procumbens* were thoroughly washed with water. The leaves were removed from any unwanted materials, plants, or plant portions and subsequently rinsed under running tap water. These were sun‐dried for 2 days and partially dried leaves were again dried in an oven at 45°C for 3 days. After drying, the materials were ground into a coarse powder using a grinding machine and then placed in an airtight container for storage (Xie et al. 2022; Cai et al. 2023).

### 2.2 Preparation of Plant Extracts

The powdered leaves of *C. cordifolia* (780 g) and *G. procumbens* (800 g) were contacted with 2000 mL of ethanol (95%) and allowed to rest for 6 h. The mixture was then filtered with Whatman No. 1 filter paper, and the process was repeated three times. The filtrate was evaporated by a cyclone evaporator at room temperature. A concentrated dark green ethanolic leaf extract of *Coccinia cordifolia* and *Gynura procumbens* was obtained after evaporation and further freeze‐dried to yield crude ethanolic leaf extracts (Xie et al. 2022; Cai et al. 2023; Rawal et al. 2025). Finally, two types of dark brown sticky crude extracts were reserved for further treatment of diabetic rats (Algariri et al. 2013).

### 2.3 Phytochemical Screening

Qualitative phytochemical screening of the ethanolic leaf extracts of *Coccinia cordifolia* and *Gynura procumbens* was carried out using standard procedures to identify the major classes of secondary metabolites. The extracts were screened for alkaloids (Dragendorff's test), steroidal compounds (Salkowski's test), phenolic compounds and tannins (ferric chloride test), flavonoids (lead acetate test), and saponins (froth test). The presence of each phytochemical class was confirmed by the characteristic color change or precipitate formation specific to the respective assay, following established protocols (Zhou et al. 2022; Mamun et al. 2026; Sultana et al. 2025).

### 2.4 Acute Toxicity Test

The research employed a well‐organized experimental framework by utilizing two types of extracts from *Coccinia cordifolia* and *Gynura procumbens*, along with three distinct dosage levels to evaluate acute toxicity. A total of 30 mice, equally split between the two groups, provides an adequately large sample size to derive significant conclusions from the experiment. The observation period of 14 days is appropriate for evaluating acute toxicity since it provides the opportunity to identify any immediate adverse effects. Three distinct dosage levels of 2, 1, and 0.5 g/kg were given, and the mice were carefully observed for any indications of death, notable behavioral alterations, or toxic reactions during the observation period. The maximum dosage of 2 g/kg holds particular significance as it indicates a substantial amount.

### 2.5 Experimental Animals

The study was conducted on 45 Wistar albino rats (140–200 g, 5–6 weeks) of either sex, which were purchased from the Animal Research Branch of the Bangladesh Council of Scientific and Industrial Research (BCSIR), Chattogram, Bangladesh. The animals were housed in plastic cages (4–6 rats each) under standard laboratory conditions at 27°C ± 1°C, relative humidity of 55%–65%, and light/dark cycle of 12 h/12 h. Experimental period: Quinone‐CaCl_2_ pure gelatin pellet (30% mN) diet or feeding rats were given a semi‐purified pellet diet and access to water ad libitum. The bedding materials were changed every 24 h to ensure proper hygiene.

Prior to experimentation, all animals were allowed to acclimatize to laboratory conditions. All experimental procedures were approved by the Institutional Animal Ethics Committee (IAEC) and performed accordingly (Mestry et al. 2017).

### 2.6 Streptozotocin‐Induced Antihyperglycemic Assay

Experimental diabetes was induced in overnight‐fasted Wistar rats by a single intravenous injection of freshly prepared streptozotocin (STZ, 40 mg/kg body weight) dissolved in 0.1 M citrate buffer (pH 4.5). This protocol is widely used to establish an experimental Type 1 diabetes mellitus model through selective destruction of pancreatic β‐cells. After 72 h, FBG levels were measured, and animals with FBG values > 180 mg/dL were considered diabetic and included in the study.

A total of 40 diabetic rats were randomly allocated into eight experimental groups (*n* = 5 per group). The sample size was selected based on previous pharmacological studies employing STZ‐induced diabetic rat models for preliminary evaluation of antihyperglycemic activity. The plant extracts (0.5, 1.0, and 2.0 g/kg body weight) and the reference drug glibenclamide (0.5 mg/kg body weight) were administered orally once daily for 14 consecutive days. The selected extract doses were based on the results of the acute toxicity study, which demonstrated no signs of toxicity up to 2 g/kg body weight and were consistent with doses reported in previous pharmacological investigations.

FBG levels were determined on Days 0, 7, and 14 using blood samples collected from the tail vein and analyzed with a glucometer based on the glucose oxidase–peroxidase (GOD–POD) method. The antihyperglycemic activity of the extracts was evaluated by comparing FBG levels among the diabetic control, glibenclamide‐treated, and extract‐treated groups (Brito et al. 2025; Akbarzadeh et al. 2007).

### 2.7 In Silico

#### 2.7.1 Molecular Docking Methodology

Molecular docking analysis was performed to investigate the binding affinity and interaction patterns of selected bioactive compounds against the antidiabetic target protein (PDB ID: 3FZ7) (Ashraf et al. 2021). Initially, 40 compounds identified through literature review were selected for virtual screening (Deb Roy et al. 2019; Iqbal et al. 2019). The three‐dimensional structure of the target receptor was obtained from the Protein Data Bank and prepared by removing water molecules and unwanted heteroatoms, followed by the addition of hydrogen atoms and energy minimization. The prepared receptor was saved in PDBQT format (3FZ7.minimized.pdbqt) for docking analysis. The ligand structures were retrieved from PubChem, optimized, and converted into appropriate docking formats before screening.

Molecular docking was carried out using AutoDock Vina implemented in PyRx software. The docking grid box was generated around the active binding region of the receptor with the following parameters: center coordinates *X* = 210.8004, *Y* = 221.0644, and *Z* = 159.1662, with grid dimensions of 25 × 25 × 25 Å. The docking calculation was performed with an exhaustiveness value of eight to ensure adequate conformational sampling and reliable binding prediction. Based on the obtained binding affinity scores, the top three compounds (2,5‐dioxopyrrolidine‐1‐carboximidamide, 2,4‐dimethylbenzo[h]quinoline, and actinobolin) were selected for detailed interaction analysis alongside glibenclamide as the reference drug. The docked complexes were visualized using Discovery Studio Visualizer to identify key amino acid residues and intermolecular interactions, including hydrogen bonds, van der Waals interactions, alkyl, pi‐alkyl, and pi‐sigma interactions.

#### 2.7.2 DFT Analysis

The quantum chemical calculations were performed using DFT to evaluate the electronic properties and chemical reactivity of the selected compounds. Geometry optimization and frontier molecular orbital (FMO) analysis were conducted using the appropriate computational level. The optimized structures were used to calculate HOMO, LUMO energies, HOMO–LUMO energy gap (*ΔE*), dipole moment, ionization potential, electron affinity, electronegativity, chemical potential, hardness, softness, and electrophilicity index. These global reactivity descriptors were analyzed to predict molecular stability, electron transfer capability, and potential biological interaction behavior. The molecular orbital distributions were visualized to interpret electron density localization.

### 2.8 Statistical Analysis

All experimental data were presented as mean ± standard error of the mean (SEM). One‐way analysis of variance followed by Dunnett's multiple comparison test was used for statistical analysis to obtain significance differences between control and treated groups. Statistical significance was set at *p* < 0.05. All the statistical analyses were performed with GraphPad Prism software (version 8.0, GraphPad Software Inc., San Diego, CA, USA).

## 3 Result

### 3.1 Phytochemical Group Test

A phytochemical analysis was performed on the ethanolic extracts of the leaves from *C. cordifolia* and *G. procumbens* to explore the existence of secondary metabolites. The results from the group tests on phytochemicals have been recorded in Table 1.

**TABLE 1:** Results of phytochemical group test of the extracts of *C. cordifolia* and *G. procumbens* leaves.

Test	Reagent	*C. cordifolia*	*G. procumbens*
Alkaloids	Dragendroff's	++	+++
Steroidal compounds	Chloroform and sulfuric acid	+++	++
Phenolic compound	Ferric chloride and potassium ferrocyanide	+++	+++
Flavonoids	10% lead acetate	++	++
Saponin	Froth test	++	±
Tannins	Ferric chloride	±	±

*Note:* +++ means strongly positive, ++ moderately positive, and ± indicates trace amount, respectively.

### 3.2 Acute Toxicity Test

The present study was undertaken to evaluate and compare the antihyperglycemic activities of leaf extracts of *C. cordifolia* and *G. procumbens* by using three different doses prepared from ethanolic extracts of these plants in an experimentally‐induced (Streptozotocin) animal diabetes model. The effects of the extracts were evaluated by comparing them to the reference medication Glibenclamide (GLB).

For the preparation of ethanolic extract, 780 g dried leaves powder of *C. cordifolia* and 805 g dried leaves of *G. procumbens* were used. After the extraction, these were 76.79 g of crude extract of *C. cordifolia* and 101.98 g of crude extract of *G. procumbens* . So, for ethanolic extract was obtained at 9.84% and 12.66% for *C. cordifolia* and *G. procumbens* , respectively.

An initial acute toxicity study was conducted to establish a suitable safe dose range that could be utilized for future experiments, rather than to offer comprehensive toxicity information on the extract. Acute toxicity studies conducted with doses of both plants that were doubled (1 and 2 g/kg body weight) compared to those used in antidiabetic research showed no behavioral, neurological, or autonomic alterations in mice (Table 2). No signs of illness or death were noted following 14 days of treatment with the decoctions. In a single dose administration, no negative effects were noted for the crude ethanolic extracts of both *C. cordifolia* and *G. procumbens* , suggesting that the extracts are nontoxic under the observed condition. The acute toxicity assessment showed that the test animals tolerated the extracts at the chosen dosages well, indicating their safety for additional studies.

**TABLE 2:** Acute toxicity data of *C. cordifolia* and *G. procumbens* leaves extract on Swiss Albino Mice model.

Parameters observed	Treatment			
	*Coccinia cordifolia*		*Gynura procumbens*	
	2 g/kg BW	1 g/kg BW	2 g/kg BW	1 g/kg BW
Stimulation				
Respiration	Normal	Normal	Normal	Normal
Agitation	Normal	Normal	Normal	Normal
Aggressiveness	Nil	Nil	Nil	Nil
Fur erection	Normal	Normal	Normal	Normal
Exophthalmia	Nil	Nil	Nil	Nil
Movement	Normal	Normal	Normal	Normal
Jaw movement	Normal	Normal	Normal	Normal
Convulsion	Nil	Nil	Nil	Nil
Tremors	Nil	Nil	Nil	Nil
Tail erection	Normal	Normal	Normal	Normal
Irritability	Nil	Nil	Nil	Nil
Depressor				
Static position	Normal	Normal	Normal	Normal
Dyspnea	Nil	Nil	Nil	Nil
Sleepiness	Nil	Nil	Nil	Nil
Prostration	Normal	Normal	Normal	Normal
Eye dullness	Nil	Nil	Nil	Nil
Others				
Fecal production	Normal	Normal	Normal	Normal
Diuresis	Normal	Increased	Increased	Increased
Spasms	Nil	Nil	Nil	Nil
Diarrhea	Nil	Nil	Nil	Nil
Regurgitation	Nil	Nil	Nil	Nil
Abdominal distension	Nil	Nil	Nil	Nil
Hemorrhagic spots	Nil	Nil	Nil	Nil

Abbreviation: Nil, no sign was observed.

### 3.3 Effect of *C. cordifolia* and *G. procumbens* Extracts by OGTT on Glucose Loaded Rats

Streptozotocin, known as a compound that induces diabetes, leads to a significant decrease in insulin secretion due to the destruction of β‐cells within the islets of Langerhans, which in turn causes hyperglycemia. *C. cordifolia* and *G. procumbens* exhibit effects that lower blood sugar levels, with a possible explanation for these antihyperglycemic properties being the enhanced secretion of insulin from the β‐cells in the islets of Langerhans or the release of insulin from its bound form. In other words, the antihyperglycemic effects of these plant extracts may also arise from actions similar to those of insulin.

This experiment was conducted to evaluate the antihyperglycemic efficacy of *C. cordifolia* and *G. procumbens* leaf extracts based on physiological and biochemical alteration by an experimental animal model.

The results in blood glucose level (BGL) after oral administration of *C. cordifolia* and *G. procumbens* crude ethanolic extracts are summarized in Table 2. Administration of streptozotocin increased (BGL) by about 46.96% for diabetic control (Group I) after 14 days compared to 1st day. Positive control (Group II) significantly reduced BGL after 14th day to 61.96% compared to the 1st day. Diabetic rats treated with *C. cordifolia* at doses 0.5, 1, and 2 g/kg b.w. (Groups VI–VIII) showed 43.67%, 49.21%, and 52.68% reduction of BGL, respectively, while *G. procumbens* at dose 0.5, 1, and 2 g/kg b.w. (Groups III–V) showed 43.75%, 51.73%, and 55.74% reduction in BGLs on the 14th day compared to the 1st day.

**FIGURE 1:**
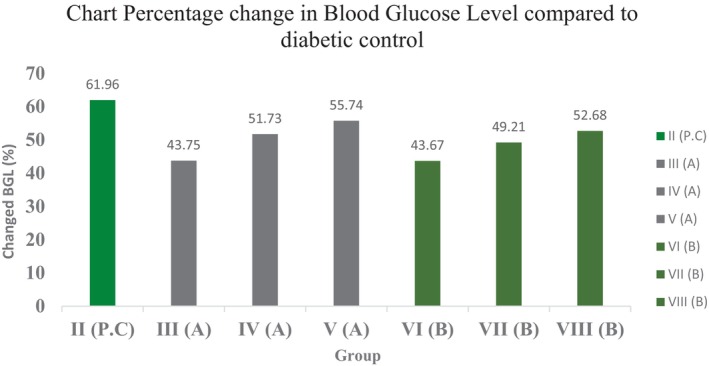
Percentage change in blood glucose level compared to diabetic control.

Among the doses tested, it is clear that 2 g/kg b.w. dose of *C. cordifolia* extract and *G. procumbens* extract showed greater efficacy 52.68% and 55.74%, respectively, against hyperglycemia. But compared to *C. cordifolia* , *G. procumbens* significantly reduced BGL. On the other hand, reference drug, glibenclamide (0.5 mg/kg b.w.) most significantly reduced BGL as 61.96% (Table 3 and Figure 1). So, the BGL reductions order is as follows:

Glibenclamide>G.procumbens>C.cordifolia.

**TABLE 3:** Comparative study of antihyperglycemic effects between *Gynura procumbens* and *Coccinia cordifolia* .

Group	Model	Dose (g/kg)	Fasting blood glucose level (mmol/L)		Changed BGL (%)
			1st day (before treatment)	14th day (after treatment)	
I	Diabetic control	—	18.10 ± 2.14***	26.60 ± 2.91	+46.96
II	Positive control	0.5	17.35 ± 0.78	6.60 ± 3.25	−61.96***
III	Ethanol extract—A	0.5	14.40 ± 1.56	8.10 ± 2.26**	−43.75***
IV	Ethanol extract—A	1.0	12.70 ± 1.80	6.13 ± 0.35	−51.73***
V	Ethanol extract—A	2.0	15.25 ± 1.63	6.75 ± 2.47	−55.74**
VI	Ethanol extract—B	0.5	17.93 ± 1.91	10.10 ± 1.83	−43.67**
VII	Ethanol extract—B	1.0	12.60 ± 2.26	6.40 ± 1.84	−49.21**
VIII	Ethanol extract—B	2.0	14.90 ± 2.69	7.05 ± 0.21	−52.68**

*Note:* Data are presented as mean ± SEM (*n* = 5). Statistical analysis was performed using one‐way ANOVA followed by Dunnett's multiple comparison test. (+): Increased BGL (%); (−): Decreased BGL (%); Diabetic control: Diabetic rats without medication; Positive control: Diabetic rats treated with Glibenclamide; Ethanol extract—A: Crude ethanol extract of *Gynura procumbens*; Ethanol extract—B: Crude ethanol extract of *Coccinia cordifolia* .

* *p* < 0.05.

** *p* < 0.01.

*** *p* < 0.001 versus the normal control group.

In the present study, the data summarized in the tables shows the antihyperglycemic efficacy of different doses of ethanolic extracts of *C. cordifolia* and *G. procumbens* , evaluated in STZ‐induced diabetic rats. Daily administration of leaf extracts from *C. cordifolia* and *G. procumbens* over a period of 14 days led to a reduction in BGLs in the Streptozotocin‐induced diabetic rats. Different doses (0.5, 1, and 2 g/kg) of both plant extracts exert different effects to control the BGL by metabolic alteration. Among the doses tested, it is clear that a 2 g/kg dose of *C. cordifolia* extract and a 2 g/kg dose of *G. procumbens* extract show greater efficacy, 52.68% and 55.74%, respectively, against hyperglycemia. The reference drug, Glibenclamide (0.5 mg/kg b.w.), most significantly reduced BGL by 61.96%. Both plant extracts showed promising antihyperglycemic effects and are comparable to Glibenclamide, suggesting their high potential to be developed as a plant‐derived antidiabetic agent.

### 3.4 Molecular Docking Analysis

The molecular docking analysis was performed to investigate the binding affinity and interaction behavior of the selected compounds against the antidiabetic target protein (PDB ID: 3FZ7). Among the screened compounds, three potential ligands (2,5‐dioxopyrrolidine‐1‐carboximidamide, 2,4‐dimethylbenzo[h]quinoline, and actinobolin) exhibited higher binding affinities compared with the standard drug glibenclamide (Table 4). The docking results demonstrated that 2,5‐dioxopyrrolidine‐1‐carboximidamide showed the highest binding affinity with a binding score of −7.5 kcal/mol, followed by 2,4‐dimethylbenzo[h]quinoline (−7.4 kcal/mol) and actinobolin (−7.2 kcal/mol), whereas glibenclamide exhibited a comparatively lower binding score of −6.3 kcal/mol (Table 4).

**TABLE 4:** Molecular docking analysis of selected bioactive compounds and reference drug glibenclamide against the antidiabetic target protein (PDB ID: 3FZ7), showing binding affinity scores and key ligand–protein interaction residues.

Activity	Ligand name	Binding score	Bond type	Binding interaction
Antidiabetic (PDB ID: 3FZ7)	2,5‐Dioxopyrrolidine‐1‐carboximidamide	−7.5	van der Waals	SER B:1202, HIS B:1203, LYS:1226
			Conventional H‐bonds	ASN B:781, PRO B:1199, TYR B:1230
	2,4‐Dimethylbenzo[h]quinoline	−7.4	van der Waals	ASN B:781, GLU B:1206, GLU B:825, SER B:1202, GLU B:1209
			Pi‐alkyl	ALA B:1205
	Actinobolin	−7.2	van der Waals	THR B:1131, GLN B:1308, ARG B:1314, GLY B:1310
			Conventional H‐bonds	SER B:1127, ASP B:1128, ILE B:1307
			Alkyl	ALA B:1311
	Glibenclamide	−6.3	van der Waals	GLU B:411, ALA A:414, GLU B:1209, GLY B:415
			Conventional H‐bonds	SER B:1202
			Pi‐alkyl	LEU B:1201, ARG B:826
			Pi‐sigma	ALA B:1205

**FIGURE 2:**
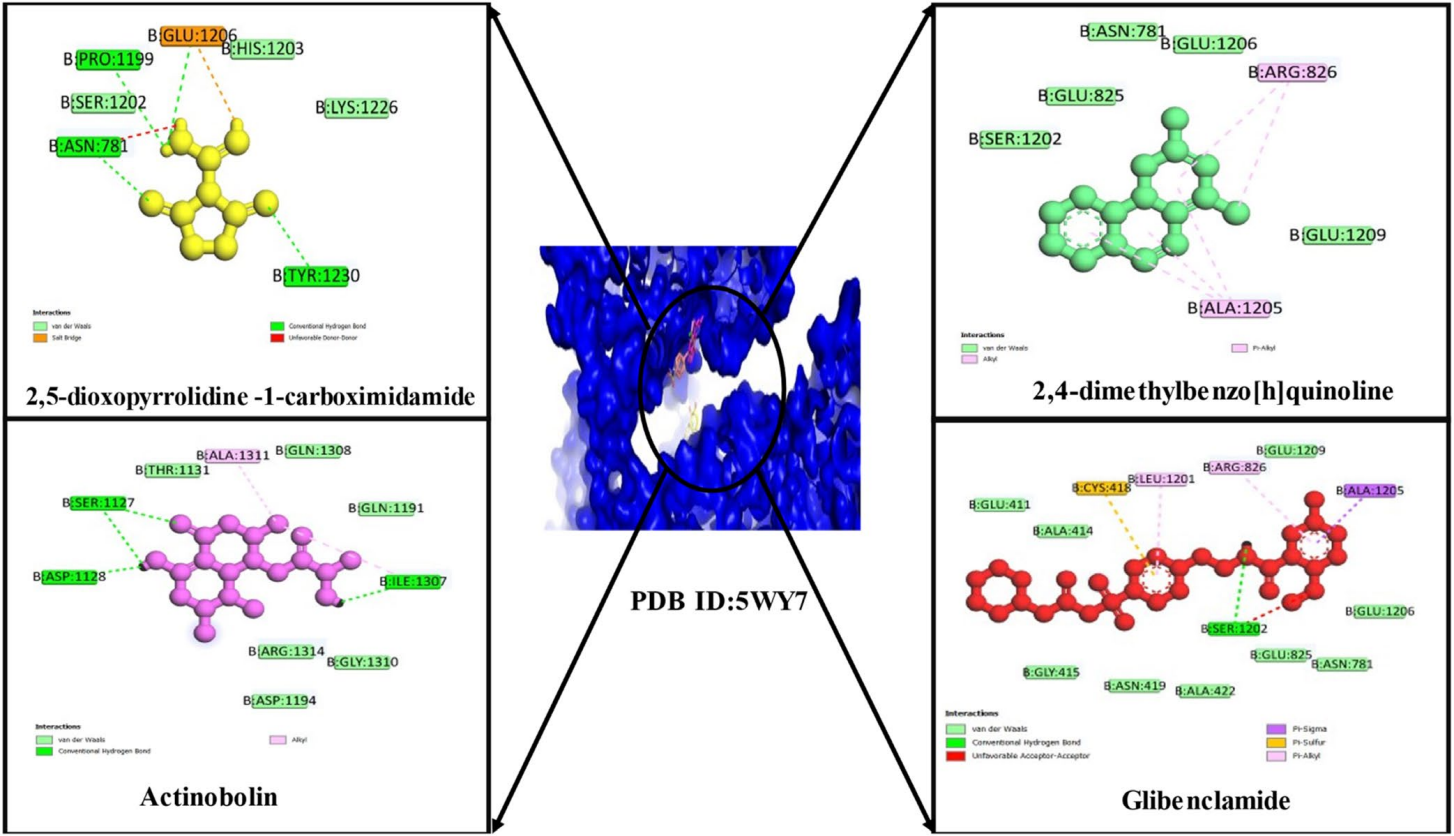
Two‐dimensional and three‐dimensional visualization of molecular docking interactions of selected ligands (2,5‐dioxopyrrolidine‐1‐carboximidamide, 2,4‐dimethylbenzo[h]quinoline, actinobolin) and reference drug glibenclamide with the antidiabetic target protein (PDB ID: 3FZ7). The figure illustrates the binding poses, hydrogen bonding, van der Waals, alkyl, pi‐alkyl, and pi‐sigma interactions involved in ligand stabilization within the active binding site.

The interaction analysis revealed that 2,5‐dioxopyrrolidine‐1‐carboximidamide formed stable interactions with important amino acid residues, including SER B:1202, HIS B:1203, and LYS B:1226 through van der Waals interactions, along with conventional hydrogen bonding interactions involving ASN B:781, PRO B:1199, and TYR B:1230 (Figure 2). The second‐ranked compound, 2,4‐dimethylbenzo[h]quinoline, showed van der Waals interactions with ASN B:781, GLU B:1206, GLU B:825, SER B:1202, and GLU B:1209, along with a pi‐alkyl interaction with ALA B:1205.

Actinobolin demonstrated a binding score of −7.2 kcal/mol and interacted with the receptor through van der Waals interactions involving THR B:1131, GLN B:1308, ARG B:1314, and GLY B:1310, while conventional hydrogen bonds were observed with SER B:1127, ASP B:1128, and ILE B:1307. Additionally, an alkyl interaction with ALA B:1311 contributed to ligand stabilization. The reference drug glibenclamide showed a lower binding affinity (−6.3 kcal/mol) and formed van der Waals interactions with GLU B:411, ALA A:414, GLU B:1209, and GLY B:415, along with hydrogen bonding with SER B:1202, pi‐alkyl interactions with LEU B:1201 and ARG B:826, and pi‐sigma interaction with ALA B:1205.

Overall, the stronger binding affinities and multiple stabilizing interactions of the selected compounds suggest their potential as promising antidiabetic candidates. The detailed 2D and 3D interaction profiles of ligand–protein complexes are presented in Figure 2, while the docking scores and interaction residues are summarized in Table 4.

### 3.5 FMO and Global Reactivity Analysis

**FIGURE 3:**
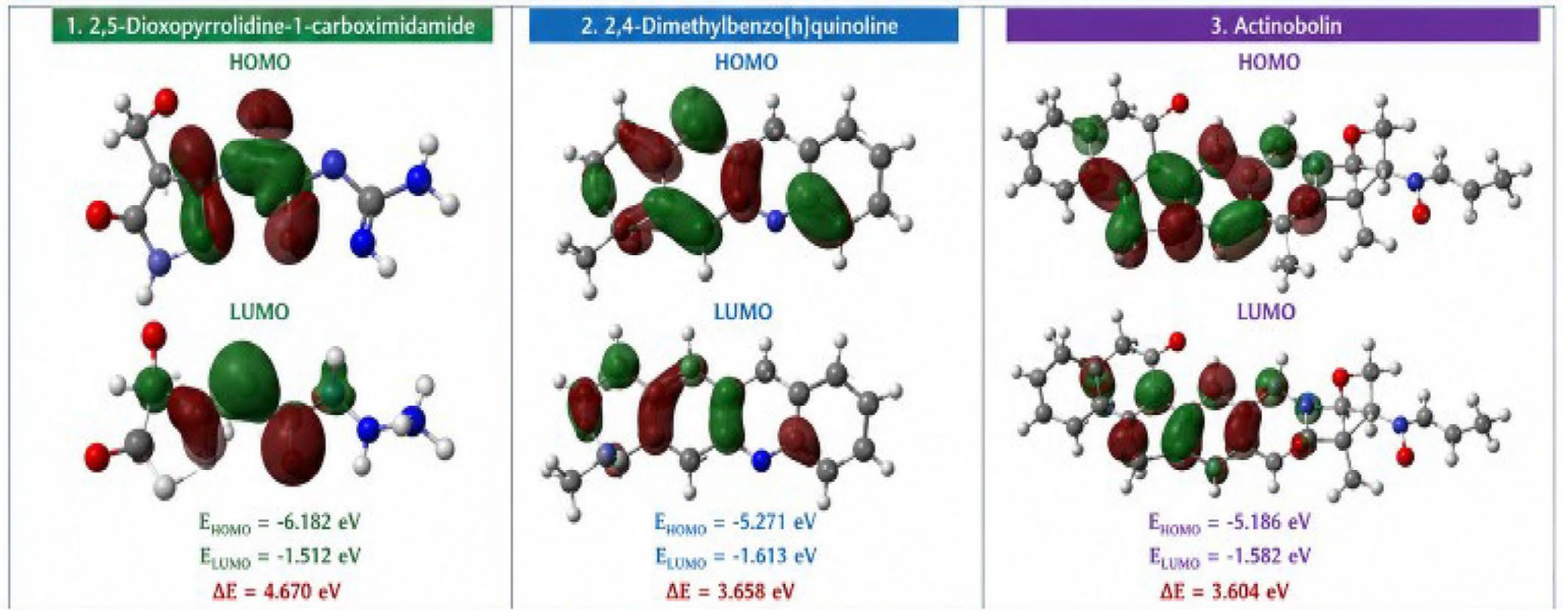
Frontier molecular orbital (FMO) analysis of the selected compounds obtained from DFT calculation. The HOMO and LUMO orbital distributions represent electron‐donating and electron‐accepting regions, respectively. The spatial distribution and energy differences between HOMO and LUMO orbitals provide insights into molecular stability, electronic transition potential, and chemical reactivity of 2,5‐dioxopyrrolidine‐1‐carboximidamide, 2,4‐dimethylbenzo[h]quinoline, and actinobolin.

The electronic properties and chemical reactivity profiles of the selected compounds were investigated using DFT analysis. The calculated FMO parameters and global reactivity descriptors are presented in Table 5, while the corresponding HOMO and LUMO orbital distributions are illustrated in Figure 3. The HOMO and LUMO energies provide valuable insights into electron‐donating and electron‐accepting abilities, respectively. The EHOMO values ranged from −6.182 to −5.186 eV, whereas ELUMO values varied between −1.613 and −1.512 eV. Among the studied compounds, actinobolin exhibited the highest HOMO energy (−5.186 eV), indicating enhanced electron‐donating capability, while 2,4‐dimethylbenzo[h]quinoline showed the lowest LUMO energy (−1.613 eV), suggesting favorable electron acceptance.

**TABLE 5:** Frontier molecular orbital (HOMO–LUMO) energies and global reactivity descriptors of the selected compounds calculated using density functional theory (DFT).

Parameters	2,5‐Dioxopyrrolidine‐1‐carboximidamide	2,4‐Dimethylbenzo[h]quinoline	Actinobolin
EHOMO (eV)	−6.182	−5.271	−5.186
ELUMO (eV)	−1.512	−1.613	−1.582
Energy gap, *ΔE* (eV)	4.670	3.658	3.604
Dipole moment (Debye)	3.126	1.847	4.532
Ionization potential, *I* (eV)	6.182	5.271	5.186
Electron affinity, *A* (eV)	1.512	1.613	1.582
Chemical potential, *μ* (eV)	−3.847	−3.442	−3.384
Electronegativity, *χ* (eV)	3.847	3.442	3.384
Chemical hardness, *η* (eV)	2.335	1.829	1.802
Chemical softness, *S* (eV^−1^)	0.428	0.547	0.555
Electrophilicity index, *ω* (eV)	3.171	3.231	3.176

*Note:* The parameters provide insights into molecular stability, electron transfer ability, polarity, and chemical reactivity.

The calculated HOMO–LUMO energy gaps (*ΔE*) were 4.670, 3.658, and 3.604 eV for 2,5‐dioxopyrrolidine‐1‐carboximidamide, 2,4‐dimethylbenzo[h]quinoline, and actinobolin, respectively (Table 3). The larger *ΔE* value of 2,5‐dioxopyrrolidine‐1‐carboximidamide indicates higher kinetic stability, whereas the lower energy gaps of actinobolin and 2,4‐dimethylbenzo[h]quinoline suggest greater chemical reactivity (Figure 3).

The dipole moment analysis revealed that actinobolin possessed the highest polarity (4.532 Debye), which may enhance intermolecular interactions. Global descriptors demonstrated that 2,5‐dioxopyrrolidine‐1‐carboximidamide had the highest electronegativity (3.847 eV) and chemical hardness (2.335 eV), reflecting greater stability. In contrast, actinobolin showed the highest softness value (0.555 eV^−1^), indicating increased molecular flexibility. The electrophilicity index ranged from 3.171 to 3.231 eV, with 2,4‐dimethylbenzo[h]quinoline showing the highest electrophilic character. Overall, DFT findings suggest that actinobolin and 2,4‐dimethylbenzo[h]quinoline possess favorable reactivity profiles, supporting their potential biological activity (Table 5 and Figure 3).

## 4 Discussion

The current study presents a detailed comparison of the antidiabetic efficacy of the ethanolic leaf extracts of two medicinal plants, *Coccinia cordifolia* and *Gynura procumbens*, via a diabetic rat model induced with streptozotocin (STZ), alongside molecular docking and DFT. It was found that both extracts caused a significant reduction in the FBG concentration in a dose‐dependent manner and no acute toxicity was observed, while computational studies have revealed a number of bioactive compounds having good binding affinity and electronic behavior with respect to their receptors (Deb Roy et al. 2019; Situmorang et al. 2025).

Phytochemical analysis revealed that the two extracts contain alkaloids, flavonoids, phenolics, steroids, saponins, and tannins, but with minor qualitative differences. Secondary metabolites are well known for their antihyperglycemic, antioxidative, and anti‐inflammatory properties. The role of flavonoids and phenolics is very significant since they are known scavengers of reactive oxygen species, reducing oxidative stress, maintaining the integrity of pancreatic beta cells, and enhancing insulin sensitivity. Alkaloids are well known to induce insulin release and regulate glucose metabolism, while saponins and tannins are known to inhibit glucose uptake from the intestines. Hence, the antihyperglycemic effect is probably due to the action of a combination of phytochemicals rather than a single active constituent (Giri et al. 2023; Shaw et al. 2010).

STZ is highly selective in the destruction of pancreatic β‐cells via DNA alkylation and oxidative stress leading to lack of insulin and hyperglycemia. Indeed, the high level of FBG seen in the control group of diabetics was indicative of the successful induction of experimental diabetes. Both plant extracts were highly effective in reducing hyperglycemia throughout the 14 days of the experiment, implying their efficacy in overcoming the metabolic dysfunctions induced by STZ. The decreasing levels of blood glucose with the increasing doses of the extracts show an evident dose‐dependent pharmacological response of the plants (Viana et al. 2004).

*Gynura procumbens* showed significantly better antihyperglycemic effects when compared to *Coccinia cordifolia* among all the plant extracts that were tested. At the maximum dose (2 g/kg), *G. procumbens* decreased FBG levels by 55.74%, while for *C. cordifolia* , it was 52.68%. In spite of the fact that glibenclamide demonstrated the best effect (61.96%), the gap between the reference substance and the high doses of the tested plant extracts was rather small. This fact confirms the high potential of the studied herbs from the pharmaceutical point of view. The significantly better activity of *G. procumbens* can be explained by a different chemical composition and presence of higher concentrations of biologically active substances which are able to increase insulin secretion, insulin sensitivity, decrease glucose production in the liver and promote glucose absorption. Such results confirm previously published research on the antidiabetic and antioxidant effects of *G. procumbens* in experimental diabetes, as well as earlier research on the hypoglycemic effects of *C. cordifolia* . The current study is valuable because it allows comparing antihyperglycemic effects of two plants under identical experimental conditions: (Pari and Latha 2005; Jamal et al. 2024).

The issue of safety is still one of the most important aspects that have to be taken into account in the development of new plant‐based medicines. According to the results of the acute toxicity test, there were no signs of death, behavioral changes, nervous and autonomic dysfunction even at the dose rate of 2 g/kg of body weight. The positive result obtained is consistent with data from other toxicological studies of both plants, which suggests the existence of a significant safety margin.

To complement the experimental findings, molecular docking analysis was performed to identify potential bioactive compounds responsible for the observed antihyperglycemic activity. Among the screened compounds, 2,5‐dioxopyrrolidine‐1‐carboximidamide, 2,4‐dimethylbenzo[h]quinoline, and actinobolin exhibited stronger binding affinities (−7.5 to −7.2 kcal/mol) than the reference drug glibenclamide (−6.3 kcal/mol). These compounds established multiple stabilizing interactions, including conventional hydrogen bonds, van der Waals interactions, alkyl interactions, and π‐alkyl interactions with important amino acid residues located within the active binding pocket of the target protein. Such interactions contribute to stable ligand–protein complex formation and suggest that these phytochemicals may effectively modulate diabetes‐related molecular targets. Although docking studies alone cannot confirm biological efficacy, the computational findings provide valuable mechanistic support for the in vivo antihyperglycemic observations and identify promising lead molecules for future drug discovery.

DFT analysis further strengthened the computational evidence by characterizing the electronic properties and chemical reactivity of the selected compounds. The calculated HOMO‐LUMO energy gaps indicated favorable molecular stability together with sufficient electronic flexibility required for biological interactions. Actinobolin demonstrated the smallest energy gap and highest softness, suggesting greater chemical reactivity and an enhanced ability to participate in electron‐transfer processes during ligand‐receptor interactions. Conversely, 2,5‐dioxopyrrolidine‐1‐carboximidamide exhibited greater chemical hardness and electronegativity, indicating higher molecular stability, while 2,4‐dimethylbenzo[h]quinoline possessed the highest electrophilicity index, reflecting an increased tendency to accept electrons during molecular recognition. Together, these electronic descriptors complement the docking results by demonstrating that the selected compounds possess physicochemical characteristics favorable for stable target binding and potential biological activity (Devi et al. 2024; Ajithkumar et al. 2020).

However, some important limitations of this research must be highlighted. For example, the investigation considered antihyperglycemic activity only on short terms and assessed changes only in FBG concentration without measuring insulin level, glycated hemoglobin concentration, lipid metabolism indices, oxidative stress markers, inflammatory mediators, and histological alterations of the pancreas. In addition, the research dealt only with crude plant extracts but not isolated phytochemicals, which does not allow defining the chemical nature of active compounds. Hence, further investigation should consider bioactivity‐guided fractionation and identification of active phytochemicals, study of their molecular mechanisms of action, chronic toxicity, pharmacokinetics, and clinical trials for evaluation of efficacy and safety of these plants.

In general, it can be stated that the combination of phytochemical screening, in vivo pharmacological investigation, molecular docking, and DFT analysis provide strong evidence for the high antihyperglycemic potential of *Coccinia cordifolia* and especially *Gynura procumbens*.

## 5 Conclusion

This current investigation confirms the significant dose‐dependent antihyperglycemic activity of the ethanolic leaves extracts of *Coccinia cordifolia* and *Gynura procumbens* in streptozotocin induced diabetic rats, which justifies their traditional uses in the treatment of DM. Both extracts had a slight variation in their glucose lowering effects in which *G. procumbens* showed a better glucose lowering effect than *C. cordifolia* ; however, both of them have shown remarkable activity when compared with the standard drug used in this experiment (glibenclamide). Phytochemical screening revealed that both the plants contain active chemical entities such as flavonoids, phenolic compounds, alkaloids, saponins, tannins and steroidal compounds which could possibly play a role in the observed effects by means of improvement in glucose homeostasis, antioxidant activities and insulin activity. Acute toxicity test confirmed that both of the extracts are safe. In addition, molecular docking identified 2,5‐dioxopyrrolidine‐1‐carboximidamide, 2,4‐dimethylbenzo[h]quinoline, and actinobolin as lead compounds having good binding affinities toward the selected antidiabetic target. The electronic properties and biological activities of the selected lead compounds are further confirmed using DFT analysis.

## 6 Limitations

Even though the results have been found to be positive, this study has its share of limitations that must be noted. To begin with, the experiment is conducted only for 14 days; thus, it is impossible to assess their long‐term effectiveness and safety. The study is focused only on the measurement of FBG level and ignores other biochemical and physiological measures, such as serum insulin, HbA1c level, lipid profile, oxidative stress markers, inflammatory cytokines, or histopathology of the pancreas. Moreover, in this experiment, only crude ethanolic extracts have been studied rather than purified bioactive phytochemicals, so there is no way of identifying the specific chemical components responsible for the antihyperglycemic effect. Besides, while the information obtained with the help of molecular docking and DFT analyses serves as predictive evidence, it needs to be validated via enzyme inhibition assays, molecular dynamic simulation, and biochemical studies related to the targets. Finally, as the results have been achieved with the help of an animal model, careful pharmacokinetic and clinical trials must be performed prior to using these extracts therapeutically.

## Author Contributions

**A. J. M. Morshed:** conceptualization, funding acquisition, writing – original draft, writing – review and editing. **Sujan Kanti Das:** conceptualization, funding acquisition, writing – original draft, writing – review and editing, supervision. **Zobayed Islam:** writing – original draft, writing – review and editing, visualization, formal analysis, software, methodology, conceptualization, investigation. **Mamuna Akter:** conceptualization, investigation, writing – original draft, validation, visualization, writing – review and editing, software, formal analysis, supervision, data curation, resources. **Muhammad Abu Bakar:** writing – original draft, writing – review and editing. **Mohammad Mostafa:** writing – original draft, writing – review and editing. **Rasheda Akter:** conceptualization, supervision, writing – original draft, writing – review and editing. **Tania Sharmin:** writing – original draft, writing – review and editing. **Mohammad Helal Uddin:** supervision, investigation, writing – original draft, writing – review and editing.

## Funding

This research was supported by an R&D Project (Memo No. 39.02.0000.011.14.111.2019/224).

## Ethics Statement

The research proposal has been reviewed and approved by the Ethical committee of BCSIR for Animal Research (ECAR). The memo number is 39.02.0000.088.99.022.23.ECAR.2025.06.

## Conflicts of Interest

The authors declare no conflicts of interest.

## Data Availability

The data that support the findings of this study are available from the corresponding author upon reasonable request.
